# Low-Threshold Optical Bistability in Detuned DBR Heterostructures Integrated with Dirac Semimetals

**DOI:** 10.3390/nano16161029

**Published:** 2026-08-19

**Authors:** Yuexiang Wu, Jiashun Hu, Weiqiang Wu, Yongxue Wang

**Affiliations:** 1 School of Integrated Circuits, Shenzhen Polytechnic University, Shenzhen 518055, China; wuyx@szpu.edu.cn; 2 School of Electronics and Communication Engineering, Shenzhen Polytechnic University, Shenzhen 518055, China; jiashunhu@szpu.edu.cn

**Keywords:** optical bistability, Dirac semimetal, optical Tamm state

## Abstract

Optical bistability plays a vital role in high-performance all-optical devices. In this work, we construct a heterostructure consisting of distributed Bragg reflectors (DBRs) and three-dimensional Dirac semimetal (3D DSM), where a 3D DSM layer is sandwiched between two DBRs with mismatched central resonant wavelengths to realize effective excitation and coupling of optical Tamm states (OTSs). Benefiting from the strong optical field confinement and intrinsic Kerr nonlinearity of 3D DSM, the proposed structure achieves a low bistability threshold of 10^5^ V/m. Moreover, the bistability performance can be tuned by modifying the Fermi level of 3D DSM and adjusting the incident angle. The proposed structure offers a promising theoretical platform for the future development of low-power terahertz bistable devices.

## 1. Introduction

Optical bistability refers to a nonlinear optical system exhibiting two distinct stable output intensities for the same input light within a certain input intensity range [1]. It has wide applications in optical switching [2], logic gates [3], optical memories [4], and biosensors [5]. Reducing the bistability threshold remains a key challenge for practical device applications. To address this, researchers have been seeking materials with higher third-order nonlinear coefficients. Early studies focused on bulk semiconductors [6], superlattices [7], and quantum wells [8]. Later, liquid crystals were also used in low-power bistable devices [9], but these systems often suffer from slow response times. In recent years, emerging nonlinear media, such as two-dimensional materials and novel photonic materials, have attracted widespread attention. Among these candidates, graphene has been widely studied due to its tunable Fermi level, strong nonlinear response, and good compatibility with terahertz and mid-infrared wavelengths. Peres et al. first theoretically discussed graphene’s bistable behavior in the terahertz range [10]. Subsequent studies explored low-threshold bistable schemes using graphene surface plasmons, defect modes, and topological edge states. For example, Dai et al. significantly reduced the bistability threshold by exciting graphene surface plasmons [11]. Zhao et al. combined graphene with local field enhancement in defect photonic crystals to lower the threshold [12]. Peng et al. realized low-threshold and tunable bistability by integrating graphene with topological edge states in one-dimensional photonic crystal heterostructures [13]. Despite its strong nonlinear potential, graphene has limitations. As an atomic-thin material, its intrinsic light–matter interaction length is limited. Moreover, practical challenges associated with the large-area growth, transfer, and integration of graphene hinder device fabrication and scalable production. Compared with graphene, 3D DSMs offer several advantages for optical bistability. They exhibit large nonlinear refractive indices [14] and, as finite-thickness films, provide a larger effective light–matter interaction volume than monolayer graphene. They also offer better device fabrication feasibility and property stability. Recently, 3D DSMs have been integrated into Fabry–Perot cavities, topological edge-state structures, and optical Tamm state architectures to achieve low-threshold, tunable bistability. For instance, Li et al. demonstrated low-threshold bistability using 3D DSM in photonic crystal Fabry–Perot cavities [15]. Long et al. proposed a 3D DSM bistable scheme based on topological edge states [16]. Wu et al. further explored combining 3D DSM nonlinearity with surface plasmon polaritons to achieve low-threshold bistability [17].

To further reduce the bistability threshold, the interaction between the optical field and the nonlinear material can be enhanced, typically by introducing localized modes such as SPPs. SPPs can confine light to subwavelength scales, significantly increasing the electric field intensity near the interface, which is particularly effective for lowering the bistability threshold. Following this approach, Sanderson et al. demonstrated optical bistability induced by nonlinear SPPs in a prism–air–graphene–dielectric structure [18]. Dai et al. achieved low-threshold terahertz bistability using graphene surface plasmons in an improved Kretschmann–Raether configuration [11]. Subsequent studies coupled SPPs with planar waveguide modes, further lowering the bistability threshold in graphene-based devices [19].

In addition to SPPs, OTSs are another important type of interfacial localized mode. They can exist at the interface between a metal and a DBR [20], or at the junction of one-dimensional photonic crystal heterostructures [21,22], and exhibit pronounced electromagnetic field localization and enhancement in the vicinity of the interface. Compared with conventional SPPs, OTSs can usually be excited directly without requiring specialized coupling components, and they support both TE- and TM-polarized waves, making them more flexible in practical device design [23]. In the realm of optical bistability, Jiang et al. demonstrated terahertz low-threshold bistability utilizing optical Tamm states within graphene–photonic crystal structures [24]. A design based on coupled graphene Tamm states was also proposed to achieve low-threshold optical bistability [25]. In 2023, a scheme for low-threshold bistability based on optical Tamm plasmon superlattices was proposed [26]. These studies demonstrate that the application of OTSs to optical bistability designs effectively reduces the threshold power.

In this work, we propose a novel 3D-DSM-embedded detuned DBR heterostructure, where a thin 3D DSM film is sandwiched between two DBRs with mismatched central wavelengths. This configuration supports coupled OTSs. Leveraging the strong field localization and excellent nonlinearity of 3D DSM, we realize low-threshold terahertz optical bistability. This design may represent a promising theoretical platform for future low-power terahertz bistable all-optical devices.

## 2. Theoretical Model and Method

**Figure 1:**
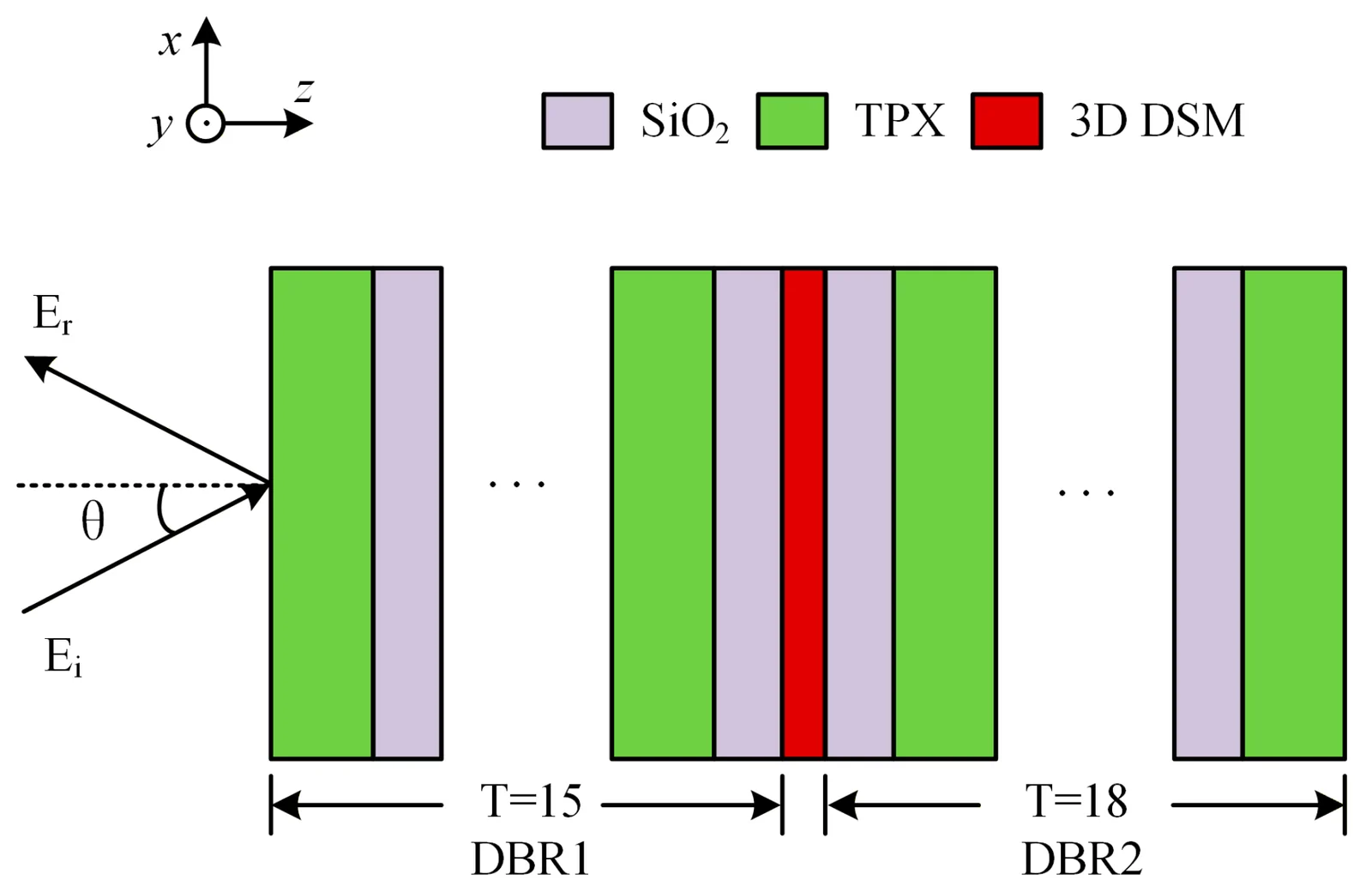
Schematic diagram of the 3D-DSM-embedded detuned DBR heterostructure.

The schematic of the proposed structure is illustrated in Figure 1. The system comprises two asymmetric DBRs and a 3D DSM layer sandwiched between them, and the adopted 3D DSM material in this work is cadmium arsenide (Cd_3_As_2_). Both DBRs are formed by alternating dielectric layers A and B, where dielectric A is SiO_2_ with a refractive index of 1.9, and dielectric B is TPX with a refractive index of 1.46. The thickness of each dielectric layer is determined by $\lambda_{ci}/4n_{j}$, where $\lambda_{ci}$ is the central wavelength of the DBR (*i* = 1, 2 corresponding to DBR1 and DBR2, respectively), and $n_{j}$ is the refractive index of the dielectric material (*j* = A for SiO_2_ and *j* = B for TPX). The left DBR1 consists of 15 periods of B/A, whereas the right DBR2 consists of 18 periods of A/B. The 3D DSM (Cd_3_As_2_) layer is denoted as layer D, with a thickness of 20 nm. In this work, all dielectric layers of TPX and SiO_2_ are approximated as lossless and nondispersive. Within the investigated THz spectral range, the linear absorption and material dispersion of these dielectric materials are comparatively weak. Our main focus lies on the nonlinear optical response originating from the 3D DSM layer, rather than linear material losses or dispersion of passive dielectric layers. Therefore, the lossless and nondispersive approximation is adopted to highlight the intrinsic bistability behavior of the 3D-DSM-based structure.

The optical transfer matrix method is used to analyze periodic photonic structures. This work only considers TE-polarized incident light, and all numerical results and conclusions in the following are restricted to TE-polarized incidence. The investigation of TM-polarized incidence is left for future work. For TE polarization, the electric field has only a *y*-component. According to the transfer matrix method, the relationship between the *y*-component of the electric field and the *x*-component of the magnetic field across the *j*-th layer is expressed as:

(1) $$\left[\begin{array}{c} E_{y}(z)\\ {-H}_{x}(z) \end{array}\right]=M_{j}\left[\begin{array}{c} E_{y}(z+d_{j})\\ {-H}_{x}(z+d_{j}) \end{array}\right]$$

Here, $j=\left\{A,B,D\right\}$, where A, B, and D denote the corresponding constituent layers in the structure. The matrix $M_{j}$ is the transfer matrix for the *j*-th layer, and its explicit form is given by:

(2) $$M_{j}=\left[\begin{array}{cc} cos(k_{jz}d_{j}) & -i\frac{\eta_{0}k_{0}}{k_{jz}}sin(k_{jz}d_{j})\\ -i\frac{k_{jz}}{{\eta_{0}k}_{0}}sin(k_{jz}d_{j}) & cos(k_{jz}d_{j}) \end{array}\right]$$

where the free-space wave number is $k_{0}=2\pi /\lambda$, with *λ* representing the wavelength of the incident light. $n_{j}$ is the refractive index of the corresponding medium. The *x*-axis and *z*-axis components of the wave vector $k_{j}=k_{0}n_{j}$ are given by $k_{jx}=$ $k_{0}n_{j}sin\theta$ and $k_{jz}=\sqrt{k_{j}^{2}-k_{jx}^{2}}$, respectively, where θ is the angle of incidence. In addition, $\eta_{0}=\sqrt{\mu_{0}/\varepsilon_{0}}$ is the wave impedance of free space, where $\mu_{0}$ and $\varepsilon_{0}$ are the permeability and permittivity of vacuum, respectively.

The refractive index of the 3D DSM layer depends on the local optical field intensity. The relationship between the refractive index and the electric field is given by [27]:

(3) $$n_{D}^{2}=n_{0}^{2}+\chi^{\left(3\right)}{\left|E\right|}^{2}$$

where $n_{0}$ is the linear refractive index of 3D DSM, and $\chi^{\left(3\right)}$ is the third-order nonlinear susceptibility.

Under the relaxation-time approximation, the linear intraband optical conductivity of the 3D DSM can be calculated using the semiclassical Boltzmann transport theory as follows [14]:

(4) $$\sigma^{\left(1\right)}=\sigma_{0}\frac{4}{3\pi^{2}}\frac{\tau }{1-i\omega \tau }\frac{{{(k}_{B}T)}^{2}}{\hbar^{2}v_{F}}[2{Li}_{2}\left(-e^{-\frac{E_{F}}{k_{B}T}}\right)+{\left(\frac{E_{F}}{k_{B}T}\right)}^{2}+\frac{\pi^{2}}{3}]$$

Here, $\sigma_{0}=e^{2}/4\hbar$, ${Li}_{2}(z)$ is the dilogarithm function, ℏ is the reduced Planck constant, T is the temperature, $k_{B}$ is the Boltzmann constant, ω is the angular frequency, $v_{F}$ = 10^6^ m/s is the Fermi velocity, $E_{F}$ is the Fermi level, and τ is the carrier relaxation time.

The third-order Kerr nonlinear optical conductivity of the 3D DSM is [14]

(5) $$\sigma^{\left(3\right)}=\sigma_{0}\frac{8e^{2}v_{F}}{5\pi^{2}\hbar^{2}}\frac{\tau^{3}}{\left(1+\omega^{2}\tau^{2})(1-2i\omega \tau \right)}\frac{1}{1+exp(-\frac{E_{F}}{k_{B}T})}$$

The Kerr susceptibility can be obtained from $\sigma^{\left(3\right)}$ as follows:

(6) $$\chi^{\left(3\right)}=\frac{i\sigma^{\left(3\right)}}{\varepsilon_{0}\omega }$$

The linear refractive index of the 3D DSM can be expressed as follows:

(7) $$n_{0}=\sqrt{1+i\sigma^{\left(1\right)}/\varepsilon_{0}\omega }$$

The refractive index of 3D DSM varies with the optical field. We adopt and improve the method presented in Ref. [28], and apply a backward recursive algorithm over sublayers for computation. In this approach, the nonlinear medium is divided into *m* thin sublayers. When *m* is sufficiently large, the refractive index within each sublayer can be approximately treated as a constant, and we initially adopt *m* = 10 for the present structure. The sublayers are numbered sequentially from left to right as 1, 2, …, *m*. Starting from the output optical field, the field intensity in the *m*th sublayer is first determined. The corresponding refractive index is then calculated from Equation (3), which allows the transfer matrix of this sublayer to be obtained according to Equation (2). The transfer matrix of the (*m*−1)th sublayer can be derived in the same manner. Repeating this procedure for all remaining sublayers, the overall transmission matrix of the 3D DSM layer can be expressed as

(8) $$M_{D}=M_{1}\cdot M_{2}\cdots M_{m}$$

The transfer matrix of the system is then as follows:

(9) $$M={{(M}_{B1}\cdot M_{A1})}^{15}\cdot M_{D}{{\cdot (M}_{A2}\cdot M_{B2})}^{18}=\left[\begin{array}{cc} m_{11} & m_{12}\\ m_{21} & m_{22} \end{array}\right]$$

The structure is assumed to be surrounded by air on both sides. The reflection and transmission coefficient of the system is derived as:

(10) $$r=\frac{\left(m_{11}+m_{12}p\right)p-\left(m_{21}+m_{22}p\right)}{\left(m_{11}+m_{12}p\right)p+\left(m_{21}+m_{22}p\right)}$$

(11) $$t=\frac{2p}{\left(m_{11}+m_{12}p\right)p+\left(m_{21}+m_{22}p\right)}$$

where $p=\frac{\sqrt{k_{0}^{2}-{{(k}_{0}sin\theta )}^{2}}}{k_{0}\eta_{0}}$. The reflectance and transmittance are $R={\left|r\right|}^{2}$ and $T={\left|t\right|}^{2}$, respectively.

In calculations, we perform a backward sweep over the output optical-field values. Equation (11) is utilized to compute the input optical field corresponding to different transmitted output optical fields. Owing to the presence of the nonlinear medium, hysteresis branches are obtained in certain regions under appropriate parameter settings via this backward-sweep approach, where a single output optical field value can correspond to multiple input optical field values. Equation (10) is further adopted to calculate the corresponding reflection coefficient and reflected optical field value.

During the calculation, we start with an initial value of *m*, i.e., the number of sublayers, and compute the reflectance *R* of the structure according to Equations (8)–(11). Afterwards, *m* is doubled and a new reflectance *R’* is recalculated. The relative deviation between the two reflectance values is defined as

(12) $$∆R=\left|\frac{R^{\prime }-R}{R}\right|$$

which serves as the convergence indicator. This procedure is repeated iteratively. As *m* increases, each sublayer becomes thinner and better approximates the actual refractive index distribution within the 3D DSM, while ∆R gradually declines. Meanwhile, as *m* increases, the relationship between the reflected optical field and the input optical field, including the optical bistability threshold, gradually converges toward the corresponding physical solution with continuously decreasing numerical variations. Convergence is deemed satisfied when the relative deviation falls below the numerical tolerance of 10^−4^.

## 3. Results and Discussion

**Figure 2:**
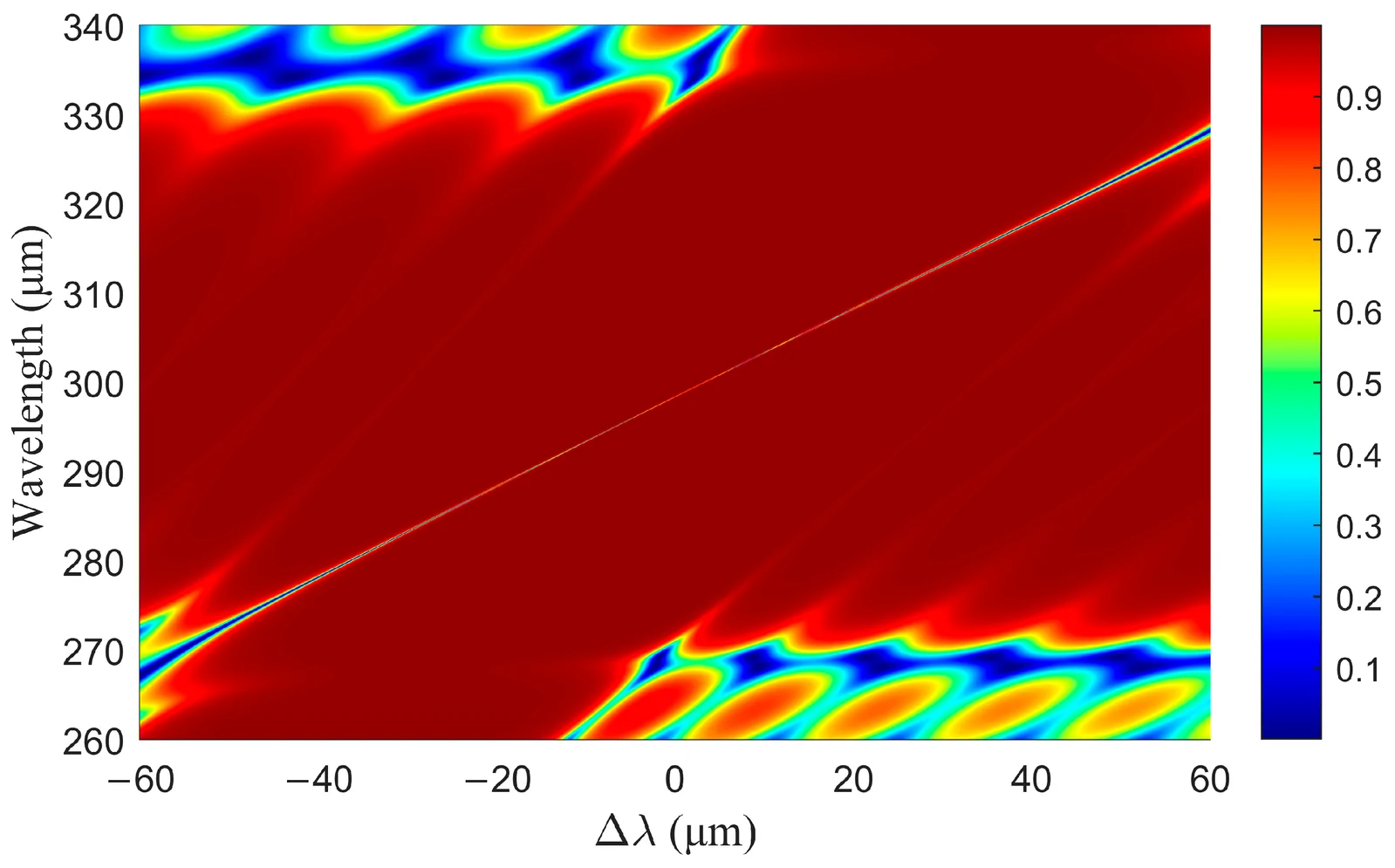
Reflectance as a function of incident wavelength and Δ*λ*.

The Fermi level of the 3D DSM is fixed at 0.06 eV, the simulation temperature is set to *T* = 300 K, and the carrier relaxation time is taken as 1 ps. The central wavelength of DBR1 is chosen as 300 μm. For convenience, the angle of the incident light is set to 10°. Under low incident light intensity, the nonlinear effect of 3D DSM can be neglected. The dependence of the reflectance on the incident light wavelength and ∆λ is depicted in Figure 2, where $∆\lambda =\lambda_{c2}-\lambda_{c1}$ is the central wavelength detuning of the two DBRs. The reflectance dip becomes deeper and broader as ∆λ increases. Since a narrower and deeper reflectance dip corresponds to a lower bistable threshold, we select a central wavelength detuning of 40 μm for DBR2, corresponding to $\lambda_{c2}=$ 340 μm.

**Figure 3:**
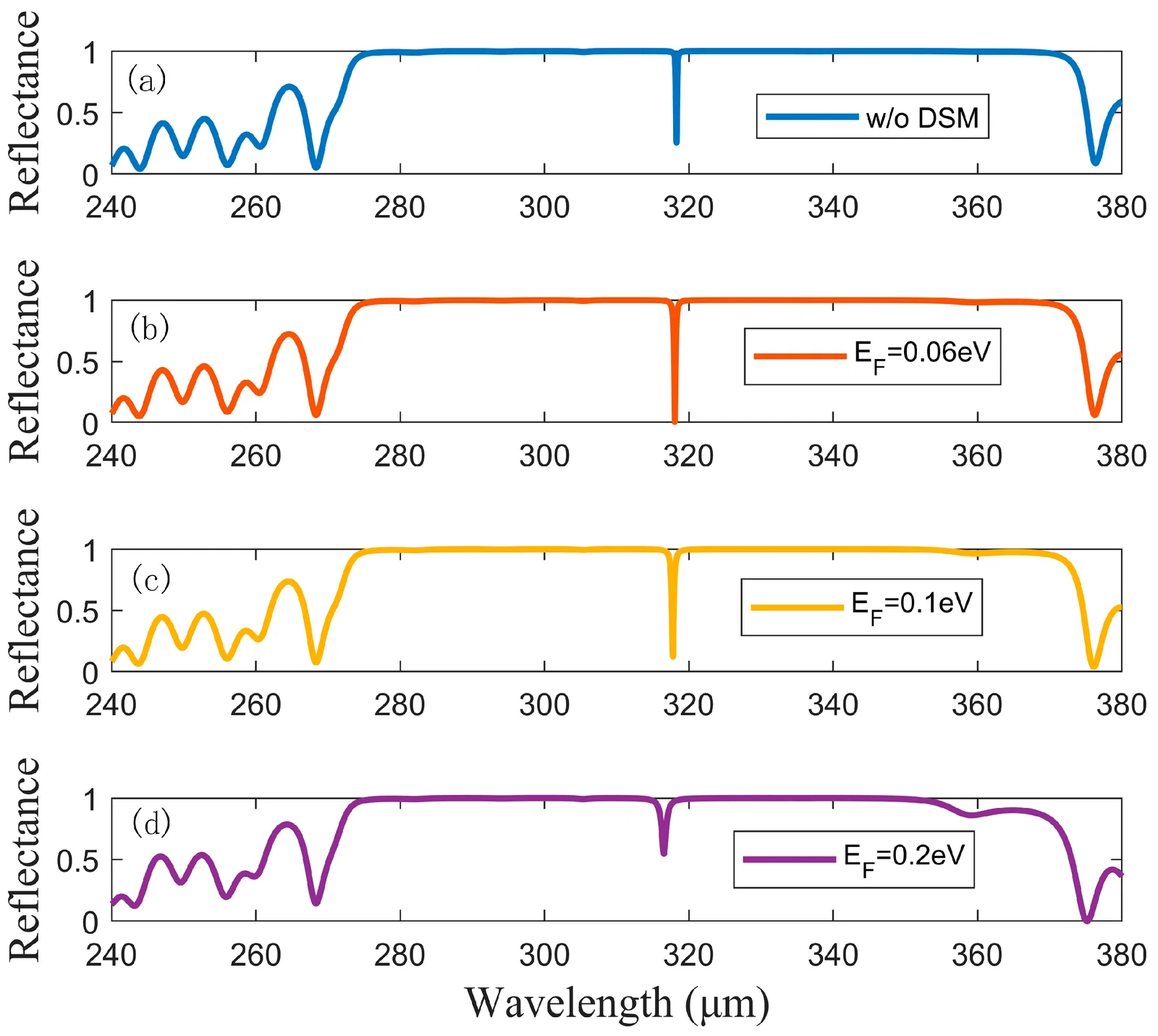
Reflectance spectra at different Fermi levels. (**a**) Without the 3D DSM; (**b**) 3D DSM with *E_F_* = 0.06 eV; (**c**) 3D DSM with *E_F_* = 0.1 eV; (**d**) 3D DSM with *E_F_* = 0.2 eV.

Subsequently, the reflectance spectrum of the structure is calculated and plotted in Figure 3. It can be seen in Figure 3a that there is a narrow reflectance dip in the structure without 3D DSM, which is induced by OTSs formed at the interface of two mismatched DBRs [21]. When a layer of 3D DSM is inserted between the two DBRs, OTSs can be excited at both the 3D DSM-DBR interfaces, resulting in mutual coupling between the OTS modes. This modifies the dip in the reflectance spectrum.

As presented in Figure 3b–d, the reflection dips broaden and become shallower with the rising Fermi level of the 3D DSM, while their corresponding resonant wavelengths exhibit a gradual blue shift. Numerical results demonstrate that the optimal reflection dip, characterized by maximum depth and a relatively narrow width, appears at 318.05 μm when the Fermi level is set to 0.06 eV. This feature is highly favorable for the generation of optical bistability.

**Figure 4:**
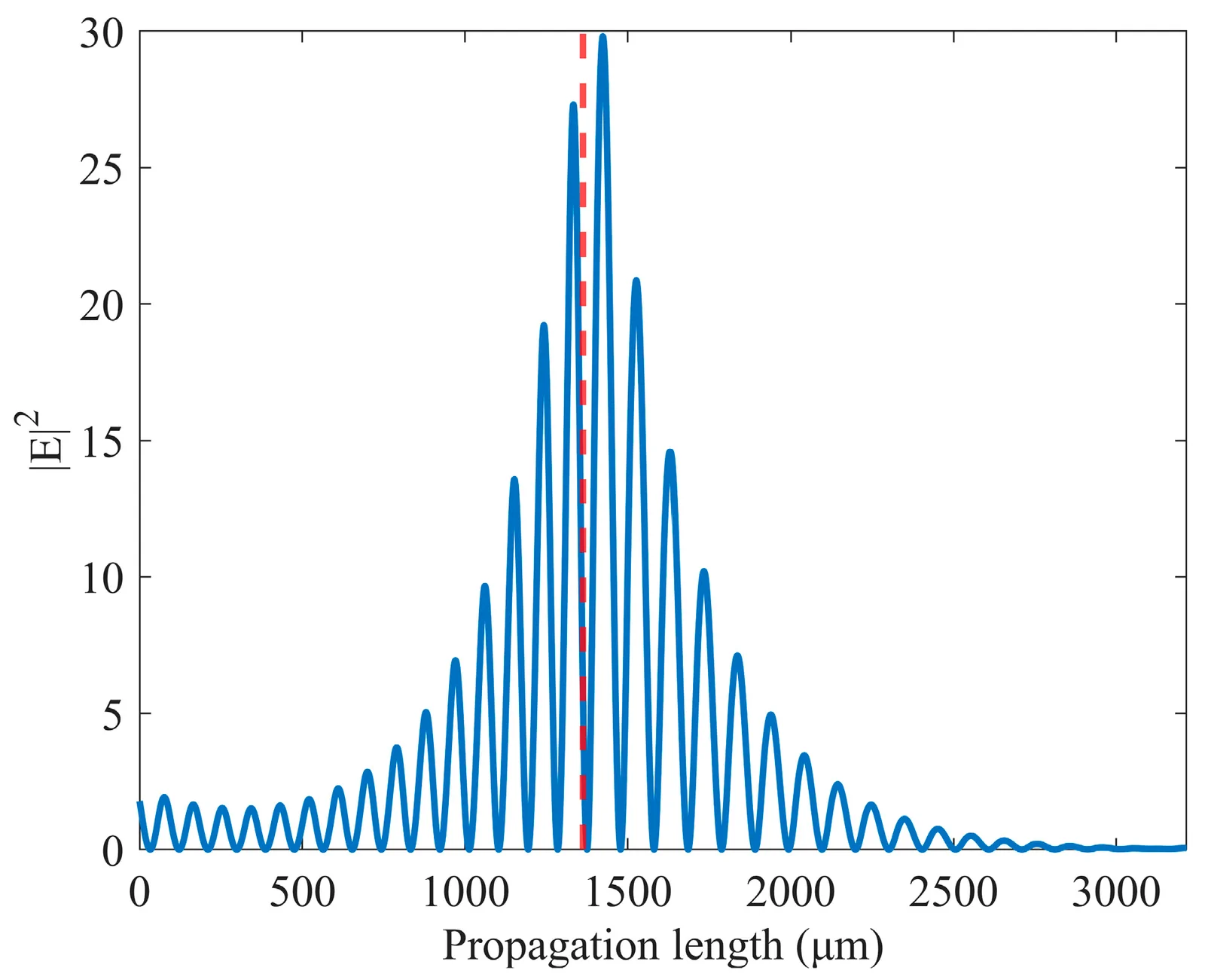
Normalized optical intensity distribution. The red dashed line indicates the position of the 3D DSM layer.

To further verify the excitation of OTSs, we calculated the optical field distribution in the structure when the Fermi level of the 3D DSM is 0.06 eV and the incident light wavelength is 318.05 μm. Other parameters were kept consistent with those in Figure 3. The optical intensity distribution shown in Figure 4 corresponds to the state at the reflectance dip in Figure 3b. In Figure 4, the incident electric field amplitude is normalized to unity, and ${\left|E\right|}^{2}$ represents the normalized optical intensity. The red dashed line in Figure 4 corresponds to the position where the 3D DSM is located. It can be observed that the optical field is strongly localized near the 3D DSM at the interface of the two DBRs and decays rapidly away from the interface toward both sides. This confirms that OTS can indeed be excited at the 3D DSM/DBR interface.

**Figure 5:**
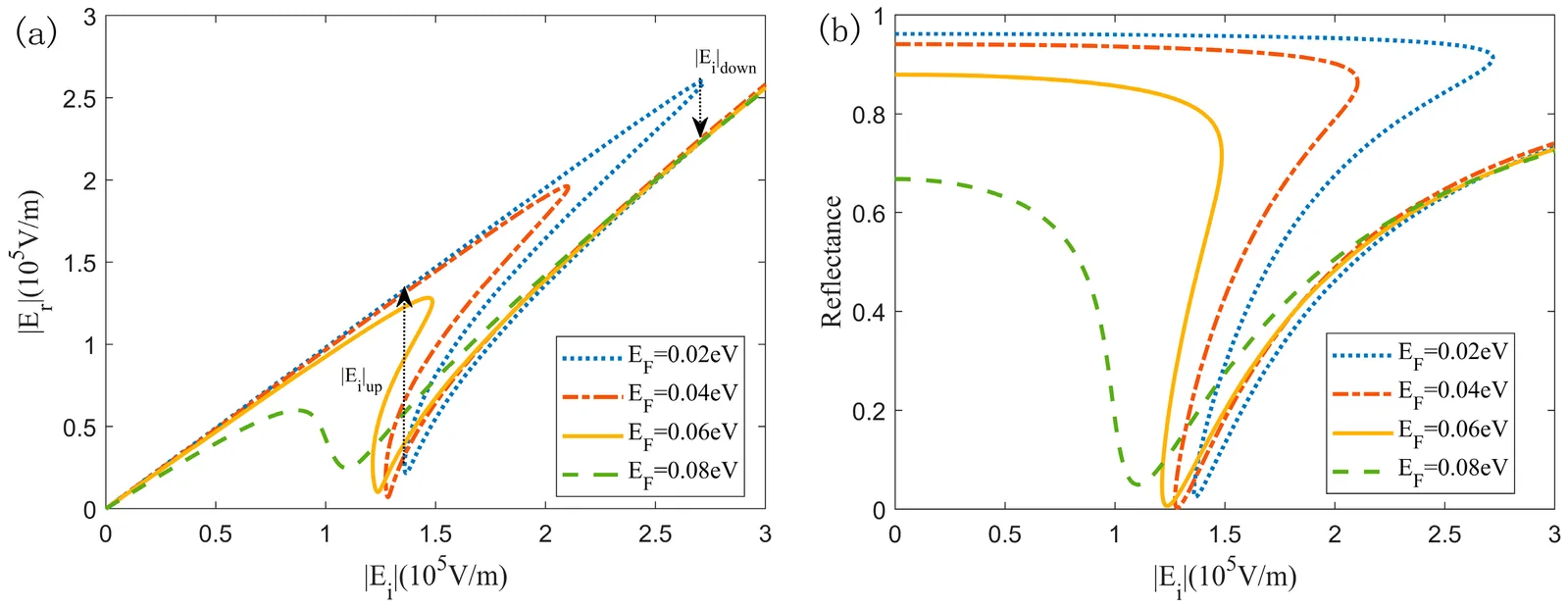
(**a**) Reflected field amplitude and (**b**) reflectance as functions of input field amplitude at different Fermi levels.

To observe optical bistability, the incident wavelength is set to 317.75 μm, which is slightly smaller than the wavelength of 318.05 μm corresponding to the reflectance dip in Figure 3b. All other parameters remain identical to those in Figure 4. The relationship between the input light and the reflected light of the structure is shown in Figure 5a. Taking the case of *E_F_* = 0.02 eV as an example, we analyze the bistable characteristics of this structure. As can be seen in Figure 5a, when the incident light field *E_i_* is weak, the reflected light field *E_r_* increases gradually with the incident light amplitude. When the incident light field reaches the switching-down threshold ${\left|E_{i}\right|}_{\mathrm{down}}$ = 2.719 × 10^5^ V/m, the reflected light field *E_r_* exhibits a sharp switch-down to a lower value, as indicated by the arrow. With a further increase in *E_i_*, *E_r_* continues to rise. Conversely, when *E_i_* is sufficiently large and is gradually reduced, the reflected light field *E_r_* switches up to a higher value at the switching-up threshold ${\left|E_{i}\right|}_{\mathrm{up}}$ = 1.359 × 10^5^ V/m. Subsequently, *E_r_* decreases continuously as *E_i_* declines. For the structure at a Fermi level of 0.02 eV, the corresponding hysteresis width is given by ${\Delta |E_{i}|\;=\;\left|E_{i}\right|}_{\mathrm{down}}-{\left|E_{i}\right|}_{\mathrm{up}}$ = 1.36 × 10^5^ V/m. The relationship between the input light and reflectance is plotted in Figure 5b. It can be observed that the values of ${\left|E_{i}\right|}_{\mathrm{down}}$, ${\left|E_{i}\right|}_{\mathrm{up}}$, and the hysteresis width $\Delta |E_{i}|$ in this figure are consistent with those in Figure 5a.

The bistable characteristics of the structure can be tuned by varying the Fermi level *E_F_* of the 3D DSM, which can be achieved by employing samples of different doping concentrations [29,30]. As shown in Figure 5a,b, both ${\left|E_{i}\right|}_{\mathrm{down}}$ and ${\;\left|E_{i}\right|}_{\mathrm{up}}$ decrease as *E_F_* increases. In particular, the reduction in ${\;\left|E_{i}\right|}_{\mathrm{up}}$ is relatively insignificant, whereas ${\left|E_{i}\right|}_{\mathrm{down}}$ shows a much more pronounced decrease, resulting in a corresponding narrowing of the hysteresis width $\Delta |E_{i}|$. At *E_F_* = 0.04 eV, ${\left|E_{i}\right|}_{\mathrm{down}}$ is 2.103 × 10^5^ V/m and ${\left|E_{i}\right|}_{\mathrm{up}}$ is 1.273 × 10^5^ V/m, corresponding to a hysteresis width of 0.83 × 10^5^ V/m. As *E_F_* increases to 0.06 eV, ${\left|E_{i}\right|}_{\mathrm{down}}$ and ${\left|E_{i}\right|}_{\mathrm{up}}$ decrease to 1.486 × 10^5^ V/m and 1.214 × 10^5^ V/m, respectively, resulting in a reduced hysteresis width of 0.272 × 10^5^ V/m. Once *E_F_* reaches 0.08 eV, however, the hysteresis effect vanishes. These results indicate that tuning the Fermi level of the 3D DSM provides a means of controlling the bistable response of the structure.

**Figure 6:**
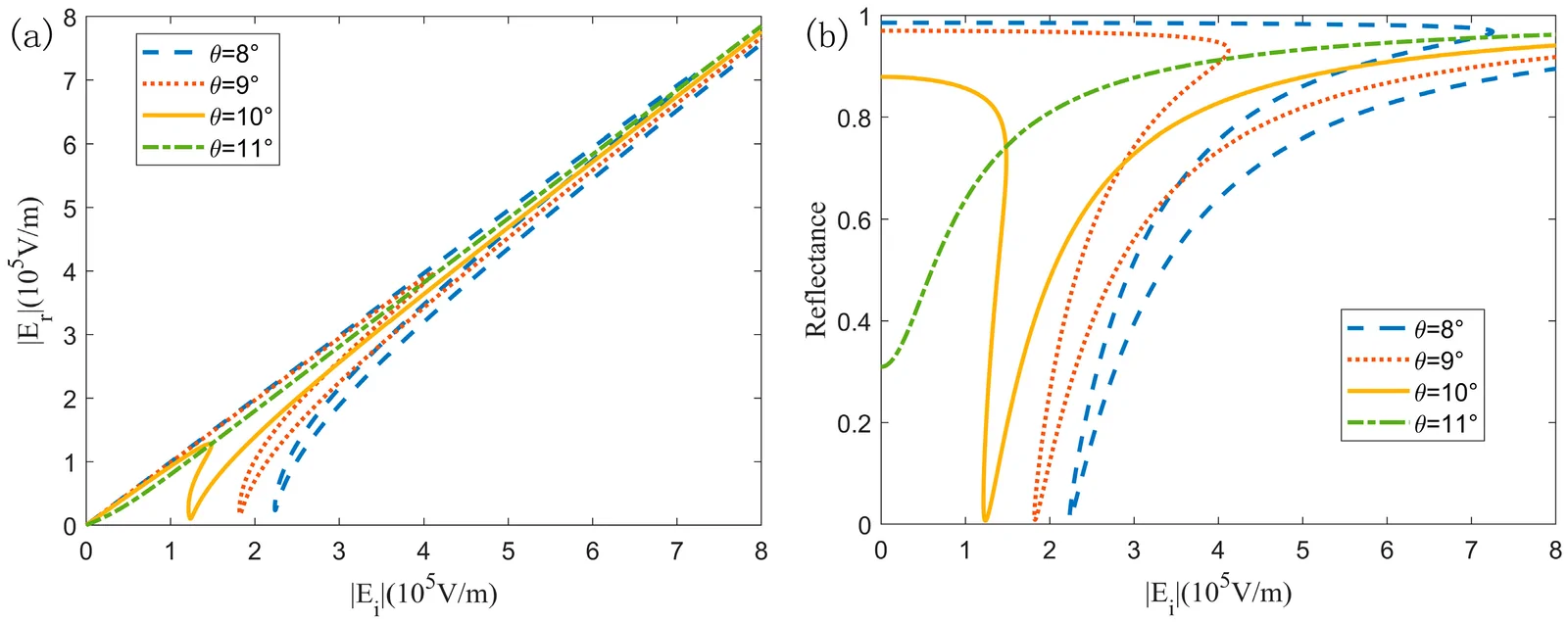
(**a**) Reflected field amplitude and (**b**) reflectance as functions of input field amplitude at different angles of incidence.

Next, the effect of the incident angle is investigated. The Fermi level of the 3D DSM is fixed at 0.06 eV, while other parameters remain the same as those used in Figure 5. Figure 6 shows how the incident angle affects the bistable behavior of the device. As seen in Figure 6a, as the incident angle decreases from 10°, both ${\left|E_{i}\right|}_{\mathrm{down}}$ and ${\;\left|E_{i}\right|}_{\mathrm{up}}$ shift to higher values, leading to a corresponding increase in the hysteresis width $\Delta |E_{i}|$. A similar trend can be observed in Figure 6b, which presents the reflectance as a function of the incident field. Specifically, at an incident angle of 9°, ${\left|E_{i}\right|}_{\mathrm{down}}$ = 4.119 × 10^5^ V/m, and ${\left|E_{i}\right|}_{\mathrm{up}}$ = 1.818 × 10^5^ V/m, yielding $\Delta |E_{i}|\;$= 2.301 × 10^5^ V/m. When the incident angle is further reduced to 8°, ${\left|E_{i}\right|}_{\mathrm{down}}$ and ${\left|E_{i}\right|}_{\mathrm{up}}$ increase to 7.251 × 10^5^ V/m and 2.237 × 10^5^ V/m, respectively, and the hysteresis width increases to 5.014 × 10^5^ V/m. However, when the angle is increased to 11°, the hysteresis loop disappears, indicating the absence of bistability. These results demonstrate that the bistable response of the structure can be flexibly tuned by varying the incident angle.

**Figure 7:**
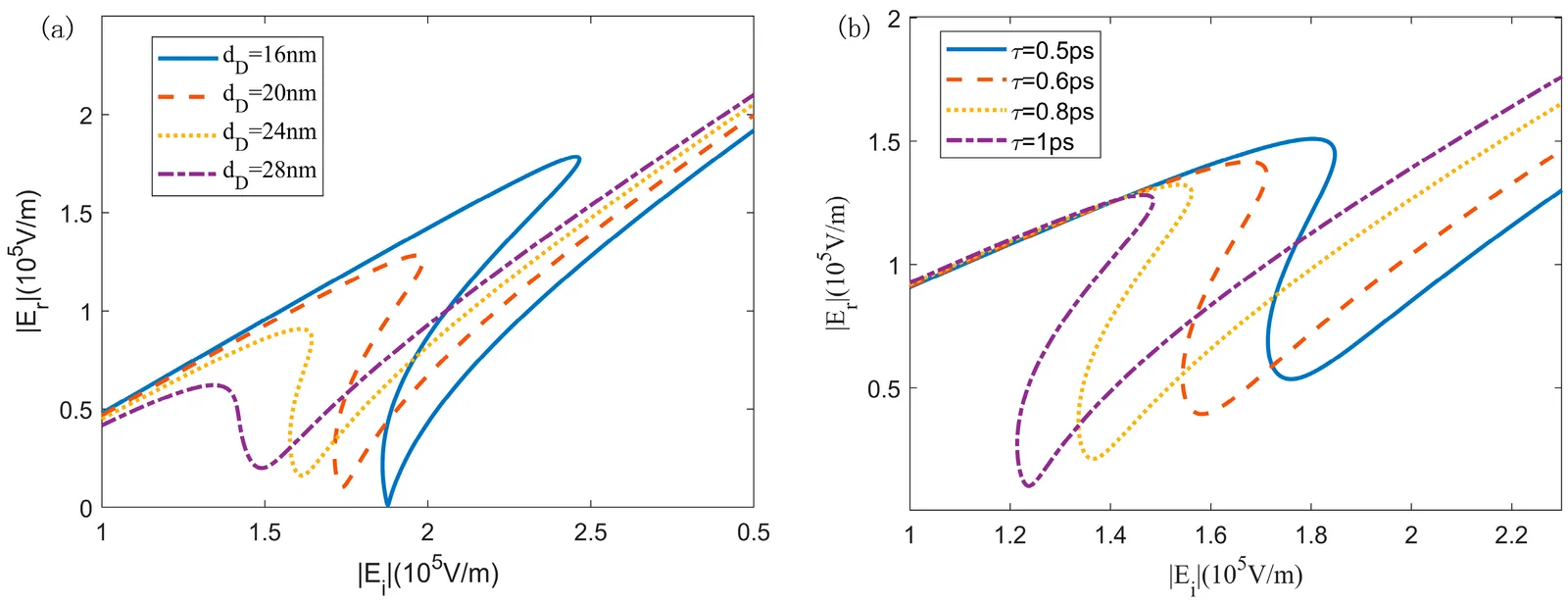
Dependence of the relationship between reflected field and incident field on (**a**) the thickness *d*_D_ and (**b**) the carrier relaxation time *τ* of the 3D DSM.

We next discuss the influences of 3D DSM thickness and carrier relaxation time *τ* on the bistable performance of the structure. As shown in Figure 7a, both the switching-up and switching-down thresholds gradually decrease with increasing 3D DSM thickness. This arises because a larger 3D DSM thickness enhances the light–matter interaction between the optical field and the nonlinear medium, thus lowering the switching thresholds. Since the switching-down threshold decreases more significantly, the hysteresis width $\Delta |E_{i}|$ is reduced accordingly. Nevertheless, the bistable behavior vanishes when the thickness reaches 28 nm. Although increasing thickness strengthens light–matter interaction, the accompanying shift of the reflectance dip (the dip position shown in Figure 3) breaks the resonant condition and suppresses bistability. Figure 7b illustrates the effect of the 3D DSM carrier relaxation time *τ* on bistability. It can be observed that, with other parameters kept constant, both the switching-up and switching-down thresholds increase gradually as *τ* decreases. A smaller carrier relaxation time corresponds to enhanced carrier scattering, which increases the optical dissipation of the Dirac semimetal. Higher loss suppresses the nonlinear optical response, thus raising the bistable switching thresholds and narrowing the hysteresis width.

**Figure 8:**
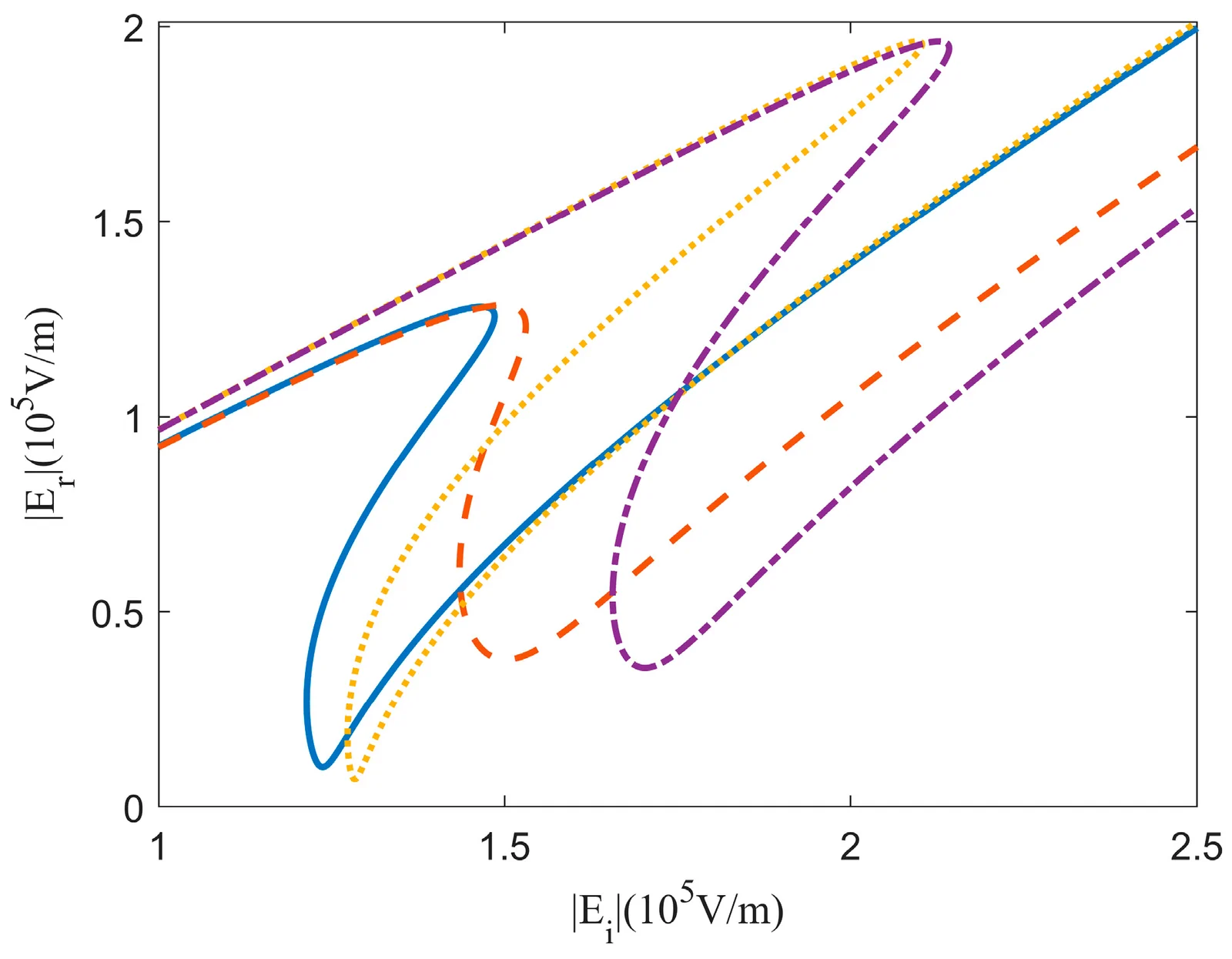
Influence of the imaginary part of 3D DSM third-order nonlinearity on the relationship between $\left|E_{r}\right|$ and $\left|E_{i}\right|$. Blue solid line: *E_F_* = 0.06 eV (imaginary part excluded); red dashed line: *E_F_* = 0.06 eV (imaginary part included); yellow dotted line: *E_F_* = 0.04 eV (imaginary part excluded); purple dash–dot line: *E_F_* = 0.04 eV (imaginary part included).

In the preceding discussion, we focused on refractive-index modulation induced by the third-order nonlinearity of 3D DSM, while the imaginary component of the third-order nonlinearity in Equation (6) was not included. Figure 8 shows the relation between output and input optical-field amplitudes when this imaginary part is incorporated. For various Fermi levels, the imaginary part scarcely affects the switching-down threshold of bistability but raises the switching-up threshold. Moreover, the deviation between the two sets of calculations at the switching-up threshold enlarges at higher Fermi levels. This reveals that the impact of the imaginary part of third-order nonlinearity, which represents the nonlinear loss arising from the 3D DSM third-order response, intensifies as the Fermi level increases.

With the above discussion, we further compare the threshold performance of our structure with that of previously reported terahertz bistable systems. Without considering the imaginary part of the third-order nonlinearity, the switching-up and switching-down thresholds of the proposed structure are 1.214 × 10^5^ V/m (1.957 × 10^7^ W/m^2^) and 1.486 × 10^5^ V/m (2.931 × 10^7^ W/m^2^), respectively, under *E_F_* = 0.06 eV and an incident angle of 10°. The values in parentheses denote the input optical intensities, which are converted from the electric-field amplitude using $I_{i}=\frac{c\varepsilon_{0}n_{0}{\left|E_{i}\right|}^{2}}{2}$, where $n_{0}$ is the refractive index of air. Compared with previously reported bistable devices in the terahertz range based on graphene [13], SPPs [19], OTSs [24], and 3D DSM [15,17], our design exhibits competitive threshold performance, especially among 3D-DSM-based bistable configurations. Notably, most reported 3D-DSM-based terahertz bistable structures require threshold intensities on the order of 10^9^ W/m^2^ or higher, while our configuration achieves considerably lower switching intensity.

## 4. Conclusions

In this work, we propose a 3D-DSM-embedded detuned DBR heterostructure capable of realizing low-threshold optical bistability in the terahertz regime. By exploiting strong field localization associated with coupled optical Tamm states and the large Kerr nonlinearity of the 3D DSM, the proposed structure achieves a low bistable switching threshold. Numerical results show that the bistable response of the device can be controlled through the Fermi level and the incident angle. Compared with graphene-based designs, the proposed structure offers a larger light–matter interaction volume and improved fabrication compatibility. This work provides a promising theoretical reference for exploring 3D-DSM-embedded detuned DBR heterostructures toward future low-power terahertz photonic devices, such as all-optical switches and optical storage elements.

## Data Availability

The original contributions presented in this study are included in the article. Further inquiries can be directed to the corresponding author.
